# Longitudinal Linkages Between Father and Mother Autonomy Support and Adolescent Problem Behaviors: Between-Family Differences and Within-Family Effects

**DOI:** 10.1007/s10964-020-01309-8

**Published:** 2020-09-02

**Authors:** Paula Vrolijk, Caspar J. Van Lissa, Susan J. T. Branje, Wim H. J. Meeus, Renske Keizer

**Affiliations:** 1 Department of Public Administration and Sociology, Erasmus University Rotterdam, P.O. Box 1738, 3000 DR Rotterdam, The Netherlands; 2 Department of Methodology and Statistics, Utrecht University, Utrecht, The Netherlands; 3 Department of Youth and Family, Utrecht University, Utrecht, The Netherlands

**Keywords:** Internalizing and externalizing problem behavior, Adolescence, Autonomy support, Random intercept cross-lagged panel model, Longitudinal

## Abstract

Despite existing evidence on negative associations between parental autonomy support and children’s internalizing and externalizing problem behavior, it is difficult to draw conclusions on the effect that parents’ autonomy support has on children’s problem behavior. This study contributed to the existing literature by unraveling the temporal ordering of parental autonomy support and adolescent problem behavior. In addition, this study examined whether these linkages differed by parent’s sex, child sex, and reporter of autonomy support. Data of 497 adolescents (mean age at T1 = 13.03 years, percentage male = 56.9) and their parents from six annual waves of the Dutch study Research on Adolescent Development And Relationships (RADAR) were used. The results showed that stable differences between families explained most linkages between autonomy support and problem behavior. Adolescents with fewer problem behaviors have fathers (both child- and parent-reported) and mothers (only child-reported) who are more autonomy supportive. The results did not differ between boys and girls. The findings suggest that prior studies may have overstated the existence of a causal effect of parental autonomy support on adolescent problem behavior.

## Introduction

Even though autonomy is a universal need across the life span, adolescence is generally considered a period in which children demand relatively more personal freedom in forming and expressing their opinions (Smetana et al. 2005). Hence, an important parental task in adolescence is supporting children’s autonomy (Joussemet et al. 2008). According to *self-determination theory*, the need for autonomy is defined as the desire to self-organize experiences and behaviors and to have activities in concordance with one’s integrated sense of self (Deci and Ryan 2000). Whereas need frustration is argued to be linked to ill-being, need satisfaction is linked to well-being. In line with this, the majority of studies on parental autonomy support are focusing on its’ positive associations with child adaptive functioning (e.g., Vasquez et al. 2016). However, the self-determination theory also posits that autonomy support protects against the development of problem behaviors, since it fosters adolescents’ coping mechanisms (Vansteenkiste and Ryan 2013). Autonomy supportive environments help building inner resources, such as emotional integration and intrinsic motivation, reducing the risk of developing problem behavior. In line with this notion, meta-analyses on parenting show that higher levels of autonomy support are related to lower levels of internalizing problems and externalizing problems, although effect sizes differ strongly between the empirical studies incorporated in the meta-analyses (McLeod et al. 2007; Pinquart 2017a, b; Yap et al. 2014). Based on existing evidence, it is difficult to make inferences about the effect that parents’ autonomy support has on children’s problem behavior. The overall aim of this study was to obtain a more nuanced understanding of linkages between autonomy support and adolescent problem behavior by unraveling the temporal ordering of parental autonomy support and adolescent problem behavior. In addition, it was examined whether these linkages differed by parents’ sex, child sex, and autonomy support reporter.

### Unraveling the Temporal Ordering of Parental Autonomy Support and Adolescent Problem Behavior

Since previous studies often employed a cross-sectional design, scholars have not been able to rule out the possibility of reverse causality, meaning that parents may adjust their level of autonomy support based on adolescents’ problem behavior. Recent longitudinal studies indeed suggest that reverse causality may be at play: when children display more internalizing and externalizing problems, parents are more likely to reduce their autonomy support (e.g., Van der Giessen et al. 2014; Van Petegem et al. 2015).

To capture how parenting affects adolescent adaptation over time, one needs to focus on the within-family effects of parental autonomy support on adolescent problem behavior. By controlling for stable between-family differences, it is possible to obtain relatively more unbiased estimates of within-family effects (Hamaker et al. 2015). Between-family differences reflect whether parents who are more autonomy supportive, also have children who show less problem behavior than parents who are less autonomy supportive. Within-family effects show whether parents who display relatively more autonomy supportive behavior than they usually do in one year, have children who subsequently show less problem behaviors than usual (or vice versa). For instance, research on parental support showed that maternal support not only positively predicted adolescents’ emotion regulation, but that higher levels of adolescents’ emotion regulation also predicted higher levels of future maternal support (Van Lissa et al. 2019).

The importance of differentiating between between-family differences and within-family effects is shown in a recent study on the relation between maternal autonomy support and adolescents’ social anxiety symptoms (Nelemans et al. 2020). When not separating within-family variance from between-family differences, the researchers did not find significant relations between autonomy support and social anxiety. However, by differentiating between between-family differences and within-family effects, they found that even though socially anxious adolescents had mothers who reported lower levels of autonomy support, mothers reported that they became more autonomy supportive after increases in social anxiety symptoms. In a different study, comparable results were found for the associations between privacy invasion and adolescent secrecy. Parental privacy invasion was positively related to adolescent secrecy on the between-family level, while higher levels of adolescent secrecy predicted future lower levels of parental privacy invasion at the within-family level (Dietvorst et al. 2018). These findings illustrate that when between-family differences and within-family effects are in opposite direction, there is a possibility of not finding any effects (or biased effects) when they are blended. There is, however, a lack of studies on parental autonomy support and adolescent problem behaviors that look into both between-family differences and within-family effects (Boele et al. 2019).

### Differences Between Fathers and Mothers

This study differentiates between paternal and maternal autonomy support, given that there are theoretical arguments to expect that in particular fathers’ autonomy support affects adolescents’ problem behavior. The *father-child activation relationship theory* posits that while mothers play an important role in children’s need to be calmed and secured, the *father-child activation relationship* plays an important role in children’s exploration of the outside world by satisfying children’s need to be stimulated, to overcome limits, and to learn to take chances (Paquette 2004). Fathers, more than mothers, may demand their children to express and think over their ideas, encourage them to take initiative, and teach them that it is okay to disagree with each other. By promoting their autonomy, fathers facilitate the process of increase in children’s exploration of the outside world and becoming more agentic. It may therefore be especially important for children’s development when fathers support children’s autonomy and give them confidence in exploring the outside environment. In contrast, when in particular fathers do not encourage the autonomy of the child, the father may give a signal that the outside world is a dangerous place, which the child is unable to handle. As a result, the child will be at risk for anxiety symptoms (Bögels and Phares 2008). So far, empirical studies that have tested the father-activation relationship are scarce. The studies that have been conducted often involved a convenience sample of solely fathers, which makes it impossible to assess whether this role is unique to fathers (see for an exception; Volling et al. 2019).

With respect to differences between fathers and mothers in the relation between autonomy support and child outcomes, empirical findings are equivocal. In line with ideas from the father-child activation relationship, research based on younger children (4.5 years) showed that fathers’, and not mothers’, encouraging and accepting behavior of children’s decisions, ideas, and emotions, was related to less internalizing problems (Van der Bruggen et al. 2010). Recent research on the relation between parenting and emotion regulation in adolescence showed that perceived parental support played a role in mother-adolescent relationships, while perceived behavioral control played a role in father-adolescent relationships. These findings suggest that it is especially important for fathers to show age-appropriate parenting and challenge adolescents to explore the outside environment (Van Lissa et al. 2019). Other studies found that both fathers’ and mothers’ autonomy supportive parenting was related to child development (Vasquez et al. 2016). Finally, there are studies showing that especially mothers’ autonomy supportive parenting is associated with positive child outcomes (Gillet et al. 2012; Stuart Parrigon and Kerns 2016).

Many scholars emphasize the importance of research that also focuses on fathers’ autonomy supportive parenting, instead of only focusing on mothers’ autonomy support (Lansford et al. 2014; van der Kaap-Deeder et al. 2017; Vasquez et al. 2016). While self-determination theory does not suggest differences in fathers’ and mothers’ role in supporting adolescents’ autonomy, the father-activation relationship posits that fathers’ autonomy support is more important for child outcomes compared to mothers’ autonomy support. In the current article, the individual contributions of fathers’ and mothers’ autonomy support on adolescents’ internalizing and externalizing problems were investigated to put ideas derived from the father-child activation theory to the test.

### Differences Between Boys and Girls

Some research suggest that parents have a larger influence on same-sex children (Bronte-Tinkew et al. 2006; Hoeve et al. 2009). According to *social learning theory*, children are more likely to be influenced by the parent who is similar to them (Bandura 1986). In contrast, self-determination theory assumes that autonomy support is equally important to all children, irrespectively of sex. This may explain why most previous studies did not test for differences between boys and girls on the relation between autonomy support and problem behaviors. We only know of one cross-sectional study that examined the moderating effect of sex. It showed that linkages between fathers’ and mothers’ parenting (promotion of independence, overcontrol, rejection) and child anxiety were similar for boys and girls (Verhoeven et al. 2012). This study further explored the differential impact of father and mother autonomy support on adolescent boys’ and girls’ problem behavior.

### Differences Between Child and Parent Reports

Although most studies on parental autonomy support use child reports (see for a review: Vasquez et al. 2016), parents’ views may provide a useful additional perspective to assess whether parental autonomy support impacts adolescents’ problem behavior. For example, whereas previous research found effects of adolescents’ social anxiety on mother-reported autonomy support, this effect was not found for child-reported autonomy support (Nelemans et al. 2020). Further, the relation between autonomy support and internalizing problems may appear stronger when using child rather than parent reports, because more depressed children might perceive parenting practices to be more negative than they actually are (Lewinsohn and Rosenbaum 1987). In line with this, a meta-analysis showed stronger relations between autonomy support and internalizing problems in studies where children reported on autonomy support and problem behavior than when parent reports or observations were used (Pinquart 2017a). For externalizing problems, this reporter effect is less clear (Pinquart 2017b). As only using parent reports on autonomy support runs the risk of obtaining socially desirable answers (Bögels and Van Melick 2004), and research focusing on both child and parent reports is scarce, it was investigated whether results differ when using different reporters.

## Current Study

Even though self-determination theory suggests that autonomy support protects against the development of problem behaviors, it is difficult to draw conclusions about the causal linkages between parental autonomy support and adolescent problem behavior. The current study expands research on parental autonomy support by examining longitudinal associations between fathers’ and mothers’ autonomy support and adolescent internalizing and externalizing problems. The first research aim was to examine between-family associations and within-family effects of parental autonomy support with adolescent problem behaviors. On the between-family level it is expected that when parents show, on average, more autonomy support than other parents, their adolescents will display, on average, less problem behaviors than other adolescents. Further, it was expected that when parents exhibit higher levels of parental autonomy support than they usually do, this decreases future adolescent problem behavior. In addition, based on earlier empirical findings, higher levels of adolescent problem behavior than usual are expected to elicit lower future parental autonomy. The second aim was to examine whether there are differences between mothers and fathers in the strength of the associations between autonomy support and problem behavior. In line with father-activation theory, it is hypothesized that fathers’ autonomy support is more strongly related to adolescent problem behavior compared to mothers’ autonomy support. The third research aim was to explore whether child sex moderated the relations between autonomy support and adolescent problem behavior. Due to lack of empirical studies and conflicting theoretical expectations, there were no clear expectations regarding this moderating effect. Finally, the fourth research aim was to examine differences in results when using different reporters (i.e., child reports and parent reports) on autonomy support. Given previous studies, and because of rater bias, linkages between autonomy support and problem behavior were expected to be stronger when children, rather than parents, reported on autonomy support.

## Methods

### Participants

The sample consisted of 497 adolescents (56.9% boys, *M*_*age*_ at T_1_ = 13.03, *SD* = 0.46) and their mothers (*N* = 495, *M*_*age*_ at T_1_ = 44.41, *SD* = 4.45) and fathers (*N* = 446, *M*_*age*_ at T_1_ = 46.74, *SD* = 5.10) who participated in the ongoing longitudinal study Research on Adolescent Development And Relationships (RADAR-young, see https://www.uu.nl/en/research/radar), which focuses on adolescents’ social relationships, personality, identity, well-being, problem behaviors, family relationships, and peer relationships. The current study used data from questionnaires that were completed during the first six annual waves. The majority of the adolescents were of Dutch origin (95.2%), and most families were classified as having a medium or high social-economic status (89.2%). During the first wave, 84.7% of the adolescents lived with both of their parents, and approximately 14.6% of the parents was divorced. Almost all adolescents answered questions about their biological or adoptive mother. Approximately 90% of the adolescents answered questions about their biological or adoptive father, 5% about another father figure, and 5% did not answer questions about their father at all.

Sample attrition was approximately 15.5% from the first to the last wave. Families who dropped out completely did not significantly differ on adolescents’ internalizing problems, *t*(72) = 1.53, *p* = 0.130, nor on externalizing problems, *t*(489) = 0.50, *p* = 0.616, during the first wave. However, there were some differences in parental autonomy support measured at Wave 1. First, adolescents from families that dropped out (*M* = 3.17, *SD* = 0.46) reported less maternal autonomy support compared to adolescents who were still participating (*M* = 3.28, *SD* = 0.40), *t*(491) = −2.02, *d* = 0.27, *p* = 0.043. Second, fathers in families that dropped out (*M* = 3.11, *SD* = 0.26) reported to be less autonomy supportive compared to fathers who were still participating in the last wave (*M* = 3.26, *SD* = 0.36), *t*(62) = −3.53, *d* = 0.49, *p* = 0.001. No differences were found for child-reported paternal autonomy support and mother-reported maternal autonomy support.

### Measurements

#### Autonomy support

To measure parental autonomy support, this study used the scale ‘balanced relatedness’, which describes the tolerance for different opinions and ideas (Shulman et al. 1997). This seven-item questionnaire was answered by parents on their own behavior towards their child and by adolescents about their father and mother separately, assessing the extent to which respondents felt parents accepted the opinions, wishes, and needs of the adolescents. Respondents could answer on a four-point scale ranging from (1) *absolutely disagree* to (4) *absolutely agree*. Example items are: “I think it is right that my child sometimes disagrees with me”/“My father/mother thinks it is right to sometimes disagree with him/her” or “I encourage my child’s suggestions”/“My father/mother encourages my suggestions”. For each wave and each separate report, the mean of the seven items was calculated to get a total score on paternal and maternal autonomy support. Construct validity, convergent validity, and test-retest reliability of the instrument was proved in earlier studies (Shulman et al. 1997; Van der Giessen et al. 2013). The scale had a good reliability for every reporter on each wave, Cronbach’s alpha ranging from *α* = 0.79 to *α* = 0.90 (see Appendix A for all Cronbach’s alpha’s per reporter).

#### Internalizing problems

Adolescents were asked about their depressive and anxiety symptoms separately to indicate adolescents’ internalizing problems. Depressive symptoms were measured using the *Reynolds Adolescent Depression Scale 2nd* *edition* (RADS-2; Reynolds 2002). This 23-item questionnaire consists of three subscales, that is dysphoric mood (8 items, such as “I feel like crying”), negative self-evaluation (8 items, such as “I feel I am bad”), and somatic complaints (7 items, such as “I am tired”). Adolescents reported on a four-point scale ranging from (1) *almost never* to (4) *most of the time*. The average score of the 23 items was calculated, to assess adolescents’ depressive symptoms. Previous research showed internal consistency, test-retest reliability, and validity (Reynolds 2002). The scale showed a good reliability for each wave, Cronbach’s alpha ranging from *α* = 0.93 to *α* = 0.95.

Anxiety symptoms were measured using the *Screen for Child Anxiety Related Emotional Disorders* (SCARED; Birmaher et al. 1997). This 38-item questionnaire assesses the occurrence of anxiety symptoms on a three-point scale: (0) *almost never*, (1) *sometimes*, and (2) *often*. Adolescents reported on five scales, including: panic disorder symptoms (13 items, such as “I’m afraid of having anxiety or panic attacks”), generalized anxiety disorder symptoms (9 items, such as “I worry if others will like me”), separation anxiety disorder symptoms (8 items, such as “I worry something bad might happen to my parents”), social phobia symptoms (4 items, such as “I feel shy with people I don’t know well”), and school anxiety symptoms (4 items, such as “I worry about going to school”). The mean score of the 38 items was computed to get an overall anxiety score. Earlier research showed good psychometric properties (Hale et al. 2011). For all waves, the scale has a good reliability, Cronbach’s alpha ranging from *α* = 0.91 to *α* = 0.94.

Depressive symptoms and anxiety symptoms were highly correlated within all waves (*r* = 0.66 to *r* = 0.78) (see Appendix B for all concurrent associations between depressive symptoms and anxiety symptoms). Since these two constructs were strongly related, it was decided to compute the mean of the normalized scores (proportion of maximum scaling; Little 2013) on depressive and anxiety symptoms to assess adolescent internalizing problems.

#### Externalizing problems

To assess externalizing problems, adolescents reported on the *Youth Self Report* (YSR; Achenbach 1991). This 30-item questionnaire consists of items on delinquent behavior (11 items, such as “I steal at home”), and aggressive behavior (19 items, such as “I get in many fights”). Adolescents were asked to indicate these behavior for the last six months and could answer on a three-point scale: (0) *almost never*, (1) *sometimes*, and (2) *often*. To assess externalizing problems, the sum of these 30 items was calculated. The scale showed good reliability, Cronbach’s alpha ranging from *α* = 0.87 to *α* = 0.91. Previous research showed adequate reliability and validity (Verhulst et al. 1997).

### Data Analysis

**Fig. 1:**
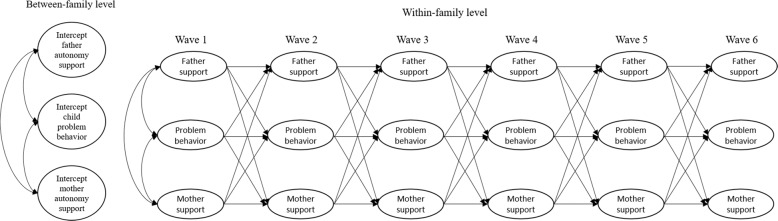
Simplified graphical representation of four random-intercept cross-lagged panel models that show the longitudinal linkages between: (1) child-reported autonomy support and internalizing problems, (2) child-reported autonomy support and externalizing problems, (3) parent-reported autonomy support and internalizing problems, and (4) parent-reported autonomy support and externalizing problems

Mplus Version 8.0 was used to ran two random intercept cross-lagged panel models (Hamaker et al. 2015) assessing the linkages between parental autonomy support and internalizing problems (separated for child versus parent reports on autonomy support) and two random intercept cross-lagged panel models assessing the linkages between parental autonomy support and externalizing problems (separated for child versus parent reports on autonomy support) (see Fig. 1). A Bonferroni-corrected significance equal to *α* = 0.05/4 = 0.012 (two-sided) was applied, since four different models were used to test for linkages between parenting and problem behavior.

The Intraclass Correlation Coefficient (ICC) of all variables was calculated. The ICC of internalizing problems was 0.64, indicating that 64% of the variance was due to stable between-family differences and 36% was due to fluctuations over time, or residual variance. For externalizing problems, 59% of the variance was explained by differences between families and 41% by over time fluctuations within families. With respect to child-reported autonomy support, 56% of the variance in father autonomy support and 60% of the variance in mother autonomy support was within-family variance. Finally, concerning parent-reported autonomy support, 41% of the variance in father autonomy support and 66% of the variance in mother autonomy support was due to fluctuations over time, or variance within families. In conclusion, a substantial part of the variance in the variables of interest was due to over time fluctuations within families. The ICC’s might also indicate that it is relevant to account for between-family differences.

To compute the four final models, first, traditional cross-lagged panel models were tested. Second, for reasons of parsimony and because no specific hypotheses were formulated about differences in relations across time, all within-time correlations, stability pathways, and cross-lagged coefficients over time were constrained, which in all cases improved model fit indices. Third, the cross-lagged panel models were extended to random-intercept cross-lagged panel models. Controlling for between-family differences led to improved model fit indices. Fourth, multi-group models were computed. The random intercepts (overall mean level) of autonomy support and problem behaviors, and the associations between autonomy support and problem behavior, were freed between boys and girls. Next, per path, child sex was explored as a possible moderator doing Wald tests. For each model, 13 Wald tests were calculated to check for differences between boys and girls. To test whether there were differences in the strength of the associations between autonomy support of mothers and fathers and problem behavior, five more Wald tests were used. To correct for multiple testing, an adjusted significance level equal to *α* = 0.05/18 = 0.003 was applied for Wald tests. Finally, paths were constrained to be equal when they were not significantly different between boys and girls.

Models were compared by checking AIC and BIC (lower values represent better models), the RMSEA (below 0.05 indicates good model fit), and the CFI and TLI (above 0.90 indicates adequate model fit, above 0.95 indicates good model fit). Dummy coded socio-economic status (1 = medium to high SES) and age of the child were used as control variables in the final model. First, SES was expected to be positively associated with average levels of autonomy support (e.g., Van Holland De Graaf et al. 2018), and negatively associated with adolescent problem behavior (e.g., Martin et al. 2010). Second, age of the child may have a negative effect on autonomy support (e.g., Gillet et al. 2012), a positive effect on internalizing problems, and a negative effect on externalizing problems (e.g., Bongers et al. 2003). Therefore, SES was included on the between-family level, and adolescent’s age was included at every wave on the observed variables. All final models had good model fit (see Tables 1 and 2). Hawkins test showed evidence of non-normality, but there was no evidence that data was not Missing Completely at Random, Jamshidian and Jalal’ s non-parametric MCAR test, *p* = 0.666. Full Information Maximum Likelihood (FIML) was used to handle missing data. Finally, since the data were not normally distributed, Full Information Maximum Likelihood with Robust estimates (MLR) was used. This procedure provides reliable estimates when the assumption of normality has been violated.

**Table 1:** Model fit indices for child-reported internalizing problems

Model	*χ*^2^	Df	Scf	AIC	aBIC	RMSEA	CFI	TLI	Δ*χ*^2^ *p*
Child-reported autonomy support									
1. Unconstrained CLPM	320.03	90	1.31	−124.93	−22.51	0.07	0.92	0.86	
2. Constrained CLPM	388.81	138	1.32	−124.75	−71.98	0.06	0.91	0.90	0.017
3. Single group RI-CLPM	168.51	132	1.32	−403.88	−344.91	0.02	0.99	0.99	<0.001
4. Sex mod. RI-CLPM	503.25	308	1.26	−479.00	−406.58	0.05	0.93	0.93	<0.001
5. Sex mod. Constrained RI-CLPM	524.85	320	1.26	−476.94	−416.94	0.05	0.93	0.93	0.041
6. Final + controls	605.00	386	1.22	−456.16	−390.98	0.05	0.93	0.93	
Parent-reported autonomy support									
1. Unconstrained CLPM	669.35	90	1.14	−2136.23	−2033.81	0.11	0.83	0.72	
2. Constrained CLPM	759.56	138	1.11	−2151.91	−2099.15	0.10	0.82	0.80	0.006
3. Single group RI-CLPM	171.27	132	1.08	−2797.87	−2738.90	0.02	0.99	0.99	<0.001
4. Sex mod. RI-CLPM	487.41	308	1.11	−2849.75	−2777.33	0.05	0.95	0.95	<0.001
5. Sex mod. Constrained RI-CLPM	508.57	320	1.10	−2854.32	−2794.32	0.05	0.94	0.95	0.042
6. Final + controls	585.54	386	1.08	−2793.11	−2727.93	0.05	0.94	0.94	

*CLPM* cross-lagged panel model, *RI-CLPM* random intercept cross-lagged panel model, *Sex mod.* moderated by child’s sex, *Constrained* all parameters that were not significantly different between boys and girls are constrained, *aBIC* adjusted BIC, *Δχ*^*2*^ *p* significance Chi-square difference test

**Table 2:** Model fit indices for child-reported externalizing problems

Model	*χ*^2^	Df	Scf	AIC	aBIC	RMSEA	CFI	TLI	Δ*χ*^2^ *p*
Child-reported autonomy support									
1. Unconstrained CLPM	323.19	90	1.28	21,303.15	21,405.58	0.07	0.91	0.84	
2. Constrained CLPM	380.89	138	1.34	21,304.50	21,357.26	0.06	0.90	0.89	0.037
3. Single group RI-CLPM	204.09	132	1.33	21,077.80	21,136.77	0.03	0.97	0.97	<0.001
4. Sex mod. RI-CLPM	415.04	308	1.30	21,080.19	21,152.61	0.04	0.96	0.96	0.040
5. Sex mod. Constrained RI-CLPM	434.45	321	1.30	21,077.80	21,136.77	0.04	0.96	0.96	0.107
6. Final + controls	531.11	387	1.25	20,700.17	20,764.33	0.04	0.95	0.95	
Parent-reported autonomy support									
1. Unconstrained CLPM	661.70	90	1.09	19,273.28	19,375.70	0.11	0.82	0.71	
2. Constrained CLPM	729.91	138	1.09	19,246.52	19,299.29	0.09	0.82	0.80	0.056
3. Single group RI-CLPM	187.07	132	1.04	18,660.21	18,719.18	0.03	0.98	0.98	<0.001
4. Sex mod. RI-CLPM	385.83	308	1.10	18,667.79	18,740.21	0.03	0.98	0.98	0.085
5. Sex mod. Constrained RI-CLPM	405.91	321	1.09	18,660.21	18,719.18	0.03	0.98	0.98	0.448
6. Final + controls	512.27	387	1.08	18,322.38	18,386.54	0.04	0.96	0.97	

*CLPM* cross-lagged panel model, *RI-CLPM* random intercept cross-lagged panel model, *Sex mod.* moderated by child’s sex, *Constrained* all parameters that were not significantly different between boys and girls are constrained, *aBIC* adjusted BIC, *Δχ*^*2*^ *p* significance Chi-square difference test

## Results

### Descriptives

**Fig. 2:**
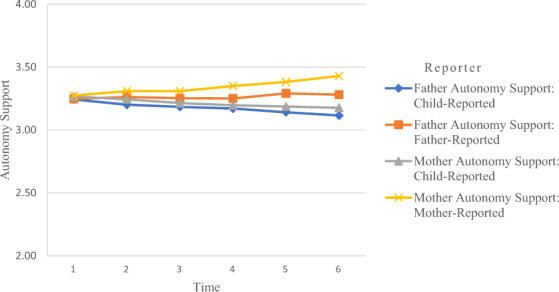
Mean level of autonomy support over time

Table 3 presents an overview of the means and standard deviations of all study variables across the six waves. Repeated measures ANOVA showed that parents and adolescents, over time, significantly differed in reported autonomy support. Figure 2 shows that, even though parent reports and child reports did not differ on the first wave, differences grew larger over time; adolescents reported less autonomy support over time, fathers reported rather stable levels of autonomy support, and mothers reported increasing levels of support over time. Adolescents reported less paternal autonomy support compared to fathers, *F*(1, 666) = 14.22, *ƞ*^2^ = 0.02, *p* < 0.001, and this difference became larger over time, *F*(5, 3330) = 6.59, *ƞ*^2^ = 0.01, *p* < 0.001. Adolescents also reported less maternal autonomy support compared to mothers, *F*(1, 742) = 31.78, *ƞ*^2^ = 0.04, *p* < 0.001, and this difference became larger over time, *F*(5, 3710) = 21.24, *ƞ*^2^ = 0.03, *p* < 0.001. Adolescents’ report of paternal and maternal autonomy support did not differ significantly. Finally, mothers reported more autonomy support compared to fathers, *F*(1, 694) = 9.15, *ƞ*^2^ = 0.01, *p* = 0.003, and this difference became larger over time, *F*(5, 3470) = 8.77, *ƞ*^2^ = 0.01, *p* < 0.001.

**Table 3:** Means and standard deviations of the measures

		Wave 1	Wave 2	Wave 3	Wave 4	Wave 5	Wave 6
		*M* (*SD*)	*M* (*SD*)	*M* (*SD*)	*M* (*SD*)	*M* (*SD*)	*M* (*SD*)
Father autonomy support (CR)							
Boys		3.25 (0.39)	3.19 (0.43)	3.18 (0.45)	3.16 (0.43)	3.14 (0.45)	3.09 (0.50)
Girls		3.24 (0.41)	3.22 (0.50)	3.19 (0.48)	3.18 (0.45)	3.14 (0.51)	3.16 (0.52)
Father autonomy support (PR)							
Boys		3.28 (0.36)	3.29 (0.36)	3.27 (0.34)	3.28 (0.35)	3.32 (0.35)	3.30 (0.38)
Girls		3.20 (0.34)	3.22 (0.34)	3.23 (0.33)	3.21 (0.32)	3.26 (0.35)	3.25 (0.35)
Mother autonomy support (CR)							
Boys		3.26 (0.40)	3.22 (0.42)	3.19 (0.42)	3.17 (0.44)	3.16 (0.43)	3.13 (0.45)
Girls		3.27 (0.42)	3.28 (0.45)	3.24 (0.47)	3.23 (0.41)	3.21 (0.45)	3.23 (0.45)
Mother autonomy support (PR)							
Boys		3.28 (0.36)	3.31 (0.37)	3.33 (0.35)	3.35 (0.40)	3.39 (0.38)	3.44 (0.40)
Girls		3.26 (0.34)	3.31 (0.35)	3.29 (0.35)	3.35 (0.38)	3.37 (0.39)	3.42 (0.39)
Internalizing problems (CR)							
Boys		0.18 (0.14)	0.12 (0.11)	0.11 (0.10)	0.12 (0.10)	0.11 (0.10)	0.13 (0.12)
Girls		0.24 (0.17)	0.20 (0.16)	0.22 (0.18)	0.22 (0.18)	0.21 (0.17)	0.22 (0.18)
Externalizing problems (CR)							
Boys		10.90 (7.45)	9.54 (8.27)	10.24 (8.18)	11.12 (8.14)	10.47 (7.63)	9.42 (7.03)
Girls		10.23 (6.75)	9.56 (8.01)	10.16 (7.74)	9.79 (7.41)	9.22 (7.07)	8.74 (7.24)

*CR* child-reported, *PR* parent-reported

Concurrent associations between child-reported paternal autonomy support and father-reported autonomy support were weak to moderate (*r* = 0.13 to *r* = 0.25). Also, concurrent associations between child-reported maternal autonomy support and mother-reported autonomy support were weak to moderate (*r* = 0.12 to *r* = 0.22). Whereas concurrent associations between child-reported paternal and maternal autonomy support were large (*r* = 0.54 to *r* = 0.67), concurrent associations between parent-reported paternal and maternal autonomy support were weak to moderate (*r* = 0.16 to *r* = 0.24). All previously mentioned zero-order correlations were significant. Concurrent associations between all measures during the first wave are represented in Table 4 (see Appendix B for concurrent associations during all waves, also separately for girls and boys in Appendix C).

**Table 4:** Concurrent associations between measures during wave 1

Variable	1	2	3	4	5	6
1. Father autonomy support: Child-reported	–	0.16*	0.58**	0.12	−0.35**	−0.27**
2. Father autonomy support: Father-reported	0.12	–	0.05	0.16*	−0.14	−0.11
3. Mother autonomy support: Child-reported	0.68**	−0.00	–	0.14*	−0.24**	−0.26**
4. Mother autonomy support: Mother-reported	0.28**	0.20**	0.22**	–	−0.07	−0.16*
5. Internalizing problems: Child-reported	−0.31**	−0.11	−0.21**	−0.16**	–	0.55**
6. Externalizing problems: Child-reported	−0.24**	−0.12	−0.19**	−0.14*	0.49**	–

All correlations below the diagonal are for boys, above the diagonal are for girls

*p < 0.05; ** *p* < 0.001

Boys and girls significantly differed on only one overall mean level. Wald tests showed that girls have on average more internalizing problems compared to boys in the child-reported autonomy support model, *χ*^2^(1) = 58.10, *p* < 0.001, and the parent-reported autonomy support model, *χ*^2^(1) = 58.21, *p* < 0.001. Therefore, the overall mean level of internalizing problems was allowed to vary between boys and girls. No other sex differences in overall mean levels of autonomy support or problem behaviors were found. There were also no significant sex differences in associations between autonomy support and problem behaviors on the between- and within-family level. Therefore, the results discussed below were similar for boys and girls.

### Between-Family Differences

**Fig. 3:**
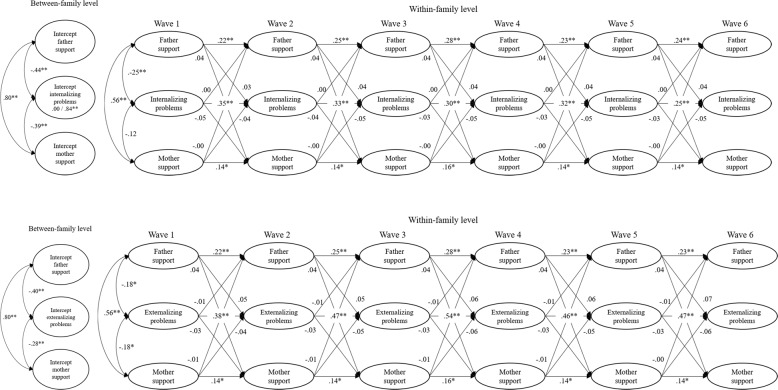
Results for child-reported models. For reasons of simplicity, associations between between-family and within-family level, concurrent correlations during Wave 2 to Wave 6, and error terms are removed. Parameters significantly different between boys and girls are represented as boys/girls (e.g., 0.00/0.84**). *p < 0.01; **p < 0.001

**Fig. 4:**
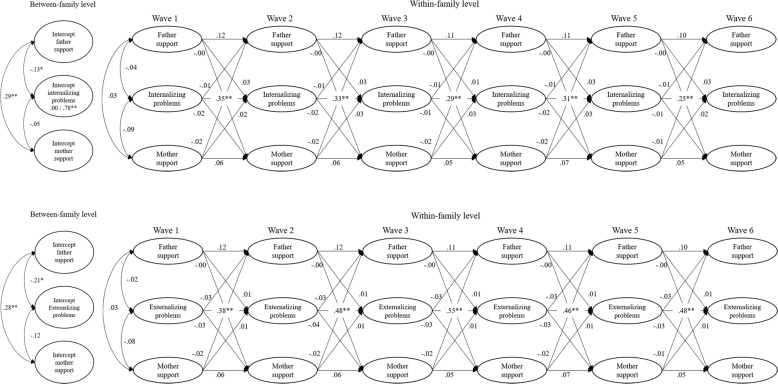
Results for parent-reported models. For reasons of simplicity, associations between between-family and within-family level, concurrent correlations during Wave 2 to Wave 6, and error terms are removed. Parameters significantly different between boys and girls are represented as boys/girls (e.g., 0.00/0.84**). *p < 0.01; **p < 0.001

Associations between overall mean levels (i.e., time-invariant family differences) showed that there is a strong relation between child-reported father autonomy support and child-reported mother autonomy support (see Fig. 3 for parameter estimates of both child-reported autonomy support models). Adolescents who, on average, reported high father autonomy support also reported that their mothers are, on average, more autonomy supportive. Also, overall mean levels of father-reported autonomy support were positively related to overall mean levels of mother-reported autonomy support (see Fig. 4 for parameter estimates of both parent-reported autonomy support models). However, this association was considerably weaker than the association in the child-reported autonomy support models.

Adolescents who showed on average more internalizing and externalizing problems, reported less paternal and maternal autonomy support. It was tested whether these between-family level associations were stronger for father autonomy support compared to mother autonomy support. Wald tests showed no significant difference for internalizing problems, *χ*^2^(1) = 1.35, *p* = 0.246, nor for externalizing problems, *χ*^2^(1) = 3.23, *p* = 0.072.

Similar results were found with respect to adolescents’ problem behaviors when fathers reported on the level of autonomy support. Fathers who reported to be more autonomy supportive had children who reported less internalizing and externalizing problems. For mothers, however, these associations were not significant. Overall mean levels of mother-reported maternal autonomy support were not significantly related to overall mean levels of adolescent problem behavior. Again, it was tested whether between-family level associations between autonomy support and adolescent problem behavior were stronger for father-reported paternal autonomy support compared to mother-reported maternal autonomy support. Wald tests showed no significant difference for internalizing, *χ*^2^(1) = 0.49, *p* = 0.485, nor for externalizing problems, *χ*^2^(1) = 1.09, *p* = 0.297. So, even though associations between autonomy support and adolescent problem behavior were negative and significant for fathers, the strength of these associations was not significantly different from the negative, but smaller, and insignificant associations for mothers.

### Within-Family Associations and Effects

#### Stability paths

Stability paths showed that child-reported autonomy support and problem behaviors were relatively stable within persons across time, indicating that respondents who scored relatively higher one year, also scored relatively higher in the next year. This might indicate a developmental trend in child-reported autonomy support and problem behavior. Parent-reported autonomy support, in contrast, was not stable within persons across time, meaning that parents who reported to be relatively higher in their autonomy support one year, were not relatively higher in their autonomy support in the next year.

#### Concurrent associations

On the within-family level, child-reported father and mother autonomy support were correlated. This correlation was not significant when parents reported on their autonomy support. Significant concurrent associations between autonomy support and problem behaviors were also only found in child-reported models. At the first wave, both internalizing and externalizing problems were negatively correlated with child-reported father autonomy support. So, higher levels of problem behaviors during the first wave were related to lower levels of father autonomy support during the first wave. Nevertheless, these relations did not continue to be significant during the other waves.

With respect to child-reported mother autonomy support, significant associations were only found with externalizing problems at the first wave. This negative association was not significant during other waves. The concurrent associations on the first wave were not significantly different for fathers’ autonomy support and mothers’ autonomy support, for internalizing, *χ*^2^(1) = 6.43, *p* = 0.011, nor for externalizing problems, *χ*^2^(1) = 0.05, *p* = 0.818. Again, even though associations were significant for fathers and not for mothers, the difference in strength was too small to be significant. Mother autonomy support was further negatively correlated with internalizing problems within Wave 2 to Wave 6. Higher levels of child-reported maternal autonomy support were concurrently associated with lower levels of internalizing problems. Again, this association was not significantly different from father autonomy support, *χ*^2^(1) = 0.30, *p* = 0.586. This result indicates that, even though there was a significant association for mothers and not for fathers, the difference in strength was not large enough to be significant.

#### Cross-lagged effects

Results showed that parents’ autonomy support did not predict adolescent problem behaviors, nor did adolescent problem behavior influence parents’ autonomy support. This was true for child-reported and parent-reported autonomy support. So, when parents displayed relatively more autonomy supportive behavior than they usually do in one year, this had no effect on adolescents’ subsequent problem behaviors. Also, when adolescents showed relatively more or less problem behaviors than they usually do in one year, this had no effect on subsequent autonomy supportive behavior of parents.

### Control Variables

Final model results were controlled for SES and age. SES had an effect on father autonomy support, irrespectively of reporter (see Appendix D for all parameters). Fathers from medium or high SES families, were more autonomy supportive compared to fathers from low SES families (*β* = 0.53 to *β* = 0.56, *p* = 0.002 for parent-reported autonomy, and *β* = 0.73 to *β* = 0.74, *p* < 0.001 for child-reported autonomy support). Next, mothers from medium or high SES families reported to be more autonomy supportive compared to mothers from low SES families (*β* = 0.40 to *β* = 0.41, *p* = 0.002). SES did not have an effect on problem behaviors. Finally, age did not have an effect on autonomy support nor problem behaviors. Results of the final models were the same when SES and age were not controlled for.

### Sensitivity Analyses

Sensitivity analyses were conducted by running models separately for fathers and mothers. Using separate models for fathers and mothers did not change the overall results. Also, equality constraints were released to check whether results would change. Overall, results remained the same, with one exception: internalizing problems were only related to lower mother-reported maternal autonomy support during two waves (*β* at T_4_ = −0.13, *p* = 0.033 and, *β* at T_6_ = −0.14, *p* = 0.025). Since these two coefficients will be assumed insignificant after correcting for multiple testing, this result should be interpreted with caution.

## Discussion

According to self-determination theory, autonomy is an important universal need that is closely linked to psychological development and functioning (Ryan et al. 2016). Parents are argued to play a central role in supporting adolescents’ need for autonomy. However, previous research was inconclusive about the effect of parental autonomy support on adolescent problem behavior. The overall aim of this study was to examine longitudinal associations between parental autonomy support and adolescent problem behavior. The findings of this study add to existing literature by differentiating between between-family differences and within-family effects, fathers’ and mothers’ autonomy support, boys’ and girls’ problem behaviors, and child-reported autonomy support and parent-reported autonomy support.

### Between-Family Differences and Within-Family Effects

The results show that, across families, adolescents with fewer internalizing and externalizing problems have fathers who are more autonomy supportive (both child- and parent-reported) and mothers who are more autonomy supportive (only child-reported). However, for both types of problem behavior, reciprocal relations at the within-family level were very weak or absent. Likewise, and in contrast to the hypothesis derived from the self-determination theory (Deci and Ryan 2000), the findings do not provide evidence for a developmental sequence, since parental autonomy support did not predict future problem behaviors or the other way around.

Instead, the linkages found between parental autonomy support and adolescent problem behavior are representing associations on the between-family level. The absence of within-family effects in this study does not rule out that within-family effects were present in earlier life stages. The between-family differences might be artifacts of within-family effects (see Van Lissa and Keizer 2020); stable characteristics of families may have been formed before the research started through a history of parent-child interactions. As autonomy support and problem behaviors were measured in adolescence, future studies may consider examining a younger age-group to check whether causal relations can be found. It could also be that between-family associations arise because autonomy support and problem behaviors are both predicted by an unknown third variable. Parents’ psychopathology, for instance, may have an effect on parenting behaviors, but also on adolescents’ psychopathology because of shared genetic factors.

The lack of within-family findings may also indicate that negative parenting, rather than positive parenting, predicts adolescent problem behaviors. For example, psychological control may frustrate adolescents’ need for autonomy, to a greater extent than a lack of autonomy support (Costa et al. 2016). Then, autonomy support may be more strongly linked to positive child outcomes, such as prosocial behavior. To examine this hypothesis, future studies may include autonomy supporting and autonomy undermining parenting behaviors in the same model.

### Differences Between Fathers and Mothers

In line with the father-child activation theory, it was expected that fathers’ autonomy support is more strongly related to adolescent problem behavior than mothers’ autonomy support (Paquette 2004). When adolescents reported on parents’ autonomy support, there was no evidence for this hypothesis; mothers’ and fathers’ autonomy support were both individually related to adolescent problem behavior (on the between-family level). In contrast, when parents reported on autonomy support, results did appear in line with the father-child activation theory (Paquette 2004). Only when fathers themselves reported more autonomy support, their children had less problem behaviors, whereas when mothers reported on their autonomy support, these associations were not found. However, like self-determination theory, father-child activation theory assumes that fathers’ autonomy support *predicts* outcomes in children. Since parental autonomy support has no causal influence on problem behaviors, and the same effects were not found for child-reported autonomy support, the findings provide little evidence for father-child activation theory. That said, the findings demonstrate that fathers’ autonomy support is related to adolescents’ problem behaviors above and beyond mothers’ autonomy support. For a clear understanding of the roles that mothers and fathers play in adolescents’ problem behaviors, future studies should incorporate both mothers’ *and* fathers’ autonomy support.

The fact that evidence was found for linkages between parent-reported autonomy support and adolescents’ problem behavior when fathers and not mothers reported on their own autonomy support, could also be explained by differences in the accuracy of their reports. Mothers tend to give a more favorable impressions of their parenting compared to fathers and adolescents (Janssens et al. 2015). If it is indeed the case that mothers, more than fathers, reported higher levels of autonomy support than they were actually giving, this will hinder finding linkages with child outcomes. Future studies on self-reports of parenting may use a responding scale sensitive to socially desirable answers to account for these possible differences (e.g., Hart et al. 2015).

### Differences Between Boys and Girls

In contrast to expectations derived from social learning theory (Bandura 1986), there were no differences between boys and girls with respect to the relations between parental autonomy support and adolescents’ problem behavior. Findings suggest that linkages between autonomy support and problem behavior are not sex-specific, and thus the provision or perception of autonomy within father-son, father-daughter, mother-son, and mother-daughter dyads are not differently related to problem behaviors. This is in line with self-determination theory, which states that the need for autonomy is equally important to every individual, irrespectively of sex.

However, it might also be the case that the research design made it less likely to detect differences between boys and girls in the investigated associations. Within the analyses, possible differences between boys and girls were examined by making between-family comparisons. By comparing siblings within the same family, researchers can more accurately check for differences between girls and boys (McHale et al. 2003). This way, it is possible to adjust for family characteristics, and get a more complete view of sex differences. Future studies are therefore encouraged to make use of sibling-designs, to assess within-family differences between boys and girls in linkages between parental autonomy support and adolescents’ problem behavior.

### Differences Between Child and Parent Reports

When adolescents reported on autonomy support, associations between parenting and adolescent problem behavior were stronger than when parents reported on their own autonomy support. This is in line with previous research showing that relations between internalizing problems and autonomy support are stronger when information comes from the same informant (Pinquart 2017a). On the one hand, this finding suggests that single source bias is present in the data. To overcome this limitation, future studies are encouraged to measure problem behaviors through another source (for instance by observations)—although it might be challenging following this suggestion, given that obtaining reliable measures of internalizing problems, like depression or anxiety, is very difficult if the reporter is not the person experiencing these issues.

On the other hand, the finding that associations between parenting and adolescent problem behavior were stronger when adolescents rather than their parents reported on parental autonomy support may simply underscore the notion that how adolescents perceive the level of autonomy support is most important for their functioning. This interpretation is in accordance with self-determination theory, which states that the degree to which children feel that their parents take their perspective and provide them with choices is satisfying their need for autonomy and therefore related to adolescents’ well-being (Ryan and Deci 2000). Earlier studies also showed that when examining the interplay between autonomy support and adolescent outcomes, child perceptions may be most important to take into consideration (e.g., Cheung et al. 2016). It is, however, important to keep in mind that for both perspectives (child and parent reports), no causal effect of autonomy support on problem behavior was found.

The results further showed that parents report higher levels of autonomy support than their children, and this discrepancy in reports becomes larger across time. This may indicate that, as children get older, their expectations of autonomy support increases, and thus their perceptions of autonomy support decreases (Gillet et al. 2012), whereas parents express that they are stable or even leveling up their autonomy support. Moreover, child reports and parent reports were only weakly or moderately related. This may reflect a lack of agreement between reporters and indicate that parents and children have different perspectives on autonomy support. Coupling these findings with the abovementioned conclusion that for adolescents’ problem behavior it matters most how they perceive their parents’ autonomy support, the results suggest that it is very important that parents in this developmental stage talk to their children about their children’s wishes for, and perceptions of, their parents’ autonomy support.

### Limitations and Future Directions

This research has some limitations that need to be mentioned when interpreting the findings. The fact that there were no within-family effects of parental autonomy support on adolescents’ problem behavior might be because of a lack of variability within problem behavior. When there is little change measured in a variable, this has an impact on the amount of variation it can explain in another variable. Even though this study focuses on adolescent problem behavior, most adolescents reported to have few or no internalizing and externalizing problems. This may have affected the lack of significant within-family findings. In addition, the sample consisted primarily of intact families with a medium or high SES. Children from high-risk families may be more sensitive to the benefits of autonomy supportive parenting compared to children from higher SES families. Positive parenting might have stronger effects in low SES families, because other resources are often missing from these children’s lives (Jeynes 2003). Future studies may therefore examine whether results are the same within a socio-economically diverse sample, which may show more variation in parenting and child outcomes. In addition, research concerning the difference between fathers and mothers in the relation between autonomy support and child outcomes is primarily focused on intact families and does not take other family structures into consideration. Future research is encouraged to investigate whether, and to what extent, linkages between fathers’ and mothers’ autonomy support and children’s problem behavior differ between intact families and divorced families.

Only effects on a long-term level (annually) were examined, whereas moment-to moment interactions occurring on a shorter time range may demonstrate different results. Future studies may consider examining autonomy support and problem behaviors on a different timescale. Especially during transitional periods, more closely spaced measurements may be needed to accurately measure changes in parenting and child outcomes, compared to more stable periods (Branje et al. 2011). As adolescence is generally considered a period in which children demand more autonomy (Smetana et al. 2005), micro-longitudinal scales (e.g., daily diaries) may, for example, show more variance in the variables under study. Since nearly all between- and within-family studies with respect to adolescent problem behaviors are examining changes on a macro timescale, future studies may also want to consider micro or meso timescales (Boele et al. 2019).

The questionnaire used for assessing autonomy support was not tapping into all autonomy supportive behaviors parents may display. The items were mainly measuring the extent to which parents are acknowledging adolescents’ feelings and providing opportunities for initiative taking. Nevertheless, a third characteristic of autonomy supportive parenting is providing rationale and explanation for rules and demands (Joussemet et al. 2008). Future studies are encouraged to use measurements that more effectively tap into these three different dimensions of parental autonomy support (e.g., Mageau et al. 2015).

Autonomy support was defined as the promotion of volitional functioning, while other researchers define autonomy support as the promotion of independence (Silk et al. 2003). This may be a limitation, since these two concepts may have a conjoint role in adolescent development (Soenens et al. 2018). Nevertheless, as opposed to the promotion of independence, the promotion of volitional functioning is more clearly linked to children’s need for autonomy (Deci and Ryan 2000), and more strongly related to adolescents’ psychological functioning (Soenens et al. 2007). Despite this, there were no effects of autonomy support on adolescent problem behavior.

## Conclusion

Because previous research had a cross-sectional design or did not take into account stable characteristics between families, it was not clear whether parental autonomy support predicts adolescent problem behavior or vice versa. By disentangling between-family differences from within-family effects, this study examined longitudinal associations between autonomy support and adolescent problem behavior. Moreover, this study examined whether these linkages differed by parent’s sex, child sex, and reporter of autonomy support. Findings show that linkages between parental autonomy support and adolescent problem behavior are largely explained by differences in stable between-family characteristics rather than in within-family effects. The results also showed that findings differ by who reports on autonomy support. The negative association between maternal autonomy support and boys’ and girls’ problem behavior was only present when children reported on parenting, whereas paternal autonomy support was linked to problem behaviors irrespectively of reporter. This study highlights the importance of using a multi-actor design, since perceptions of parenting may differ between parents and children. By using innovative methods that accurately estimate reciprocal relations within families, this study did not find any within-family effects, which suggests that prior studies have overestimated the predictive role of parental autonomy support in determining adolescent problem behavior.

## Supplementary information

Appendix
