# Supplementary material for: Longitudinal Linkages Between Father and Mother Autonomy Support and Adolescent Problem Behaviors: Between-Family Differences and Within-Family Effects
Source: J Youth Adolesc. 2020 Sep 2;49(11):2372–87. doi: 10.1007/s10964-020-01309-8 (PMC7538400; doi:10.1007/s10964-020-01309-8)
Supplement: Supplementary file 1 — Appendix [file 10964_2020_1309_MOESM1_ESM.docx]

| Wave | 1 | 2 | 3 | 4 | 5 | 6 |
| --- | --- | --- | --- | --- | --- | --- |
|  |  |  |  |  |  |  |
| **Child reports** |  |  |  |  |  |  |
| Father autonomy support | .79 | .84 | .86 | .84 | .88 | .89 |
| Mother autonomy support | .85 | .86 | .86 | .86 | .87 | .89 |
| **Parent reports** |  |  |  |  |  |  |
| Father autonomy support | .84 | .85 | .86 | .87 | .85 | .89 |
| Mother autonomy support | .85 | .87 | .86 | .89 | .89 | .90 |
| **Internalizing problems** |  |  |  |  |  |  |
| Depression | .93 | .94 | .94 | .95 | .94 | .94 |
| Anxiety | .91 | .93 | .93 | .94 | .93 | .94 |
| **Externalizing problems** |  |  |  |  |  |  |
| Youth self-report | .87 | .91 | .89 | .89 | .89 | .88 |

Appendix A

*Cronbach’s Alpha’s For All Wave*

| Appendix B.  *Concurrent Associations For All Waves*  Correlations: Wave 1 |  |  |  |  |  |  |  |  |
| --- | --- | --- | --- | --- | --- | --- | --- | --- |
| Variable | 1 | 2 | 3 | 4 | 5 | 6 | 7 | 8 |
| 1. Father autonomy support: Child-reported | 1 |  |  |  |  |  |  |  |
| 2. Father autonomy support: Father-reported | .13** | 1 |  |  |  |  |  |  |
| 3. Mother autonomy support: Child-reported | .64** | .01 | 1 |  |  |  |  |  |
| 4. Mother autonomy support: Mother-reported | .21** | .18** | .18** | 1 |  |  |  |  |
| 5. Internalizing problems | -.33** | -.14** | -.22** | -.12** | 1 |  |  |  |
| 6. Externalizing problems | -.25** | -.11* | -.22** | -.14** | .49** | 1 |  |  |
| 7. Depressive symptoms | -.34** | -.11* | -.23** | -.11* | x | .47** | 1 |  |
| 8. Anxiety symptoms | -.22** | -.20** | -.10 | -.06 | x | .38** | .66** | 1 |
|  |  |  |  |  |  |  |  |  |
| Correlations: Wave 2 |  |  |  |  |  |  |  |  |
| Variable | 1 | 2 | 3 | 4 | 5 | 6 | 7 | 8 |
| 1. Father autonomy support: Child-reported | 1 |  |  |  |  |  |  |  |
| 2. Father autonomy support: Father-reported | .22** | 1 |  |  |  |  |  |  |
| 3. Mother autonomy support: Child-reported | .61** | .18** | 1 |  |  |  |  |  |
| 4. Mother autonomy support: Mother-reported | .18** | .23** | .22** | 1 |  |  |  |  |
| 5. Internalizing problems | -.23** | -.13** | -.15** | -.09 | 1 |  |  |  |
| 6. Externalizing problems | -.31** | -.12* | -.20** | -.08 | .34** | 1 |  |  |
| 7. Depressive symptoms | -.27** | -.12* | -.19** | -.11* | x | .37** | 1 |  |
| 8. Anxiety symptoms | -.14** | -.11* | -.08 | -.05 | x | .22** | .72** | 1 |

| Correlations: Wave 3 |  |  |  |  |  |  |  |  |
| --- | --- | --- | --- | --- | --- | --- | --- | --- |
| Variable | 1 | 2 | 3 | 4 | 5 | 6 | 7 | 8 |
| 1. Father autonomy support: Child-reported | 1 |  |  |  |  |  |  |  |
| 2. Father autonomy support: Father-reported | .25** | 1 |  |  |  |  |  |  |
| 3. Mother autonomy support: Child-reported | .57** | .19** | 1 |  |  |  |  |  |
| 4. Mother autonomy support: Mother-reported | .16** | .22** | .14** | 1 |  |  |  |  |
| 5. Internalizing problems | -.22** | -.14** | -22** | -.04 | 1 |  |  |  |
| 6. Externalizing problems | -.24** | -.21** | -.24** | -.11* | .37** | 1 |  |  |
| 7. Depressive symptoms | -.26** | -.14** | -.23** | -.05 | x | .39** | 1 |  |
| 8. Anxiety symptoms | -.13** | -.12* | -.17** | -.01 | x | .28** | .78** | 1 |
|  |  |  |  |  |  |  |  |  |
| Correlations: Wave 4 |  |  |  |  |  |  |  |  |
| Variable | 1 | 2 | 3 | 4 | 5 | 6 | 7 | 8 |
| 1. Father autonomy support: Child-reported | 1 |  |  |  |  |  |  |  |
| 2. Father autonomy support: Father-reported | .17** | 1 |  |  |  |  |  |  |
| 3. Mother autonomy support: Child-reported | .67** | .14** | 1 |  |  |  |  |  |
| 4. Mother autonomy support: Mother-reported | .20** | .22** | .17** | 1 |  |  |  |  |
| 5. Internalizing problems | -.21** | -.13* | -19** | -.08 | 1 |  |  |  |
| 6. Externalizing problems | -.16** | -.10* | -.12* | -.08 | .27** | 1 |  |  |
| 7. Depressive symptoms | -.28** | -.13* | -.24** | -.11* | x | .30** | 1 |  |
| 8. Anxiety symptoms | -.10* | -.11* | -.10* | -.04 | x | .18** | .76** | 1 |
|  |  |  |  |  |  |  |  |  |
|  |  |  |  |  |  |  |  |  |

| Correlations: Wave 5 |  |  |  |  |  |  |  |  |
| --- | --- | --- | --- | --- | --- | --- | --- | --- |
| Variable | 1 | 2 | 3 | 4 | 5 | 6 | 7 | 8 |
| 1. Father autonomy support: Child-reported | 1 |  |  |  |  |  |  |  |
| 2. Father autonomy support: Father-reported | .25** | 1 |  |  |  |  |  |  |
| 3. Mother autonomy support: Child-reported | .61** | .21** | 1 |  |  |  |  |  |
| 4. Mother autonomy support: Mother-reported | .16** | .24** | .12* | 1 |  |  |  |  |
| 5. Internalizing problems | -.17** | -.05 | -27** | -.01 | 1 |  |  |  |
| 6. Externalizing problems | -.18** | -.13* | -.21** | -.04 | .31** | 1 |  |  |
| 7. Depressive symptoms | -.20** | -.07 | -.27** | -.05 | x | .33** | 1 |  |
| 8. Anxiety symptoms | -.10* | -.02 | -.22** | .04 | x | .24** | .77** | 1 |
|  |  |  |  |  |  |  |  |  |
| Correlations: Wave 6 |  |  |  |  |  |  |  |  |
| Variable | 1 | 2 | 3 | 4 | 5 | 6 | 7 | 8 |
| 1. Father autonomy support: Child-reported | 1 |  |  |  |  |  |  |  |
| 2. Father autonomy support: Father-reported | .23** | 1 |  |  |  |  |  |  |
| 3. Mother autonomy support: Child-reported | .54** | .19** | 1 |  |  |  |  |  |
| 4. Mother autonomy support: Mother-reported | .11* | .16** | .22** | 1 |  |  |  |  |
| 5. Internalizing problems | -.24** | -.09 | -.15** | .02 | 1 |  |  |  |
| 6. Externalizing problems | -.15** | -.07 | -.10* | .04 | .47** | 1 |  |  |
| 7. Depressive symptoms | -.30** | -.11* | -.21** | -.01 | x | .47** | 1 |  |
| 8. Anxiety symptoms | -.13** | -.06 | -.05 | .06 | x | .40** | .76** | 1 |

| Appendix C.  *Concurrent Associations For All Waves*  Correlations boys (below) and girls (above) Wave 1 |  |  |  |  |  |  |  |  |
| --- | --- | --- | --- | --- | --- | --- | --- | --- |
| Variable | 1 | 2 | 3 | 4 | 5 | 6 | 7 | 8 |
| 1. Father autonomy support: Child-reported |  | .16* | .58** | .12 | -.35** | -.27** | -.34** | -.23* |
| 2. Father autonomy support: Father-reported | .12 | 1 | .05 | .16* | -.14 | -.11 | -.11 | -.13 |
| 3. Mother autonomy support: Child-reported | .68** | -.00 | 1 | .14* | -.24** | -.26** | -.23** | -.13 |
| 4. Mother autonomy support: Mother-reported | .28** | .20** | .22** | 1 | -.07 | -.16* | -.05 | -.09 |
| 5. Internalizing problems | -.31** | -.11 | -.21** | -.16** | 1 | .55** | x | x |
| 6. Externalizing problems | -.24** | -.12 | -.19** | -.14* | .49** | 1 | .53** | .45** |
| 7. Depressive symptoms | -.34** | -.09 | -.25** | -.15* | x | .45** | 1 | .67** |
| 8. Anxiety symptoms | -.29** | -.22** | -.15 | -.07 | x | .43** | .64** | 1 |
| Correlations boys (below) and girls (above) Wave 2 |  |  |  |  |  |  |  |  |
| Variable | 1 | 2 | 3 | 4 | 5 | 6 | 7 | 8 |
| 1. Father autonomy support: Child-reported | 1 | .18* | .56** | .23** | -.26** | -.31** | -.33** | -.15* |
| 2. Father autonomy support: Father-reported | .27** | 1 | .18* | .23** | -.08 | -.04 | -.09 | -.06 |
| 3. Mother autonomy support: Child-reported | .65** | .20 | 1 | .29** | -.20** | -.17* | -.23** | -.15* |
| 4. Mother autonomy support: Mother-reported | .15* | .24** | .18** | 1 | -.08 | -.11 | -.12 | -.03 |
| 5. Internalizing problems | -.24** | -.14* | -.16** | -.10 | 1 | 35** | x | x |
| 6. Externalizing problems | -.30** | -.18** | -.23** | -.05 | .38** | 1 | .42** | .21** |
| 7. Depressive symptoms | -.25** | -.11 | -.20** | -.11 | x | .37** | 1 | .73** |
| 8. Anxiety symptoms | -.17** | -.13 | -.06 | -.07 | x | .27** | .64** | 1 |
|  |  |  |  |  |  |  |  |  |

| Correlations boys (below) and girls (above) Wave 3 |  |  |  |  |  |  |  |  |
| --- | --- | --- | --- | --- | --- | --- | --- | --- |
| Variable | 1 | 2 | 3 | 4 | 5 | 6 | 7 | 8 |
| 1. Father autonomy support: Child-reported | 1 | .26** | .54** | .15* | -.32** | -.32** | -.40** | -.18* |
| 2. Father autonomy support: Father-reported | .24** | 1 | .20** | .33** | -.12 | -.15* | -.13 | -.09 |
| 3. Mother autonomy support: Child-reported | .61** | .19** | 1 | .17* | -.34** | -.32** | -.37** | -.26** |
| 4. Mother autonomy support: Mother-reported | .17** | .13* | .12 | 1 | -.01 | -.19* | -.03 | .02 |
| 5. Internalizing problems | -.13* | -.16* | -.14* | -.04 | 1 | .35** | x | x |
| 6. Externalizing problems | -.18** | -.26* | -.19** | -.05 | .50** | 1 | .38** | .27** |
| 7. Depressive symptoms | -.14* | -.14* | -.13* | -.05 | x | .52** | 1 | .77** |
| 8. Anxiety symptoms | -.10 | -.16* | -.12 | -.00 | x | .39** | .70** | 1 |
| Correlations boys (below) and girls (above) Wave 4 |  |  |  |  |  |  |  |  |
| Variable | 1 | 2 | 3 | 4 | 5 | 6 | 7 | 8 |
| 1. Father autonomy support: Child-reported | 1 | .23** | .65** | .20** | -.28** | -.26** | -.35** | -.15* |
| 2. Father autonomy support: Father-reported | .13 | 1 | .14 | .20** | -.08 | -.11 | -.08 | -.07 |
| 3. Mother autonomy support: Child-reported | .69** | .15* | 1 | .14* | -.33** | -.23** | -.37** | -.23** |
| 4. Mother autonomy support: Mother-reported | .19** | .24** | .19** | 1 | -.08 | -.10 | -.10 | -.04 |
| 5. Internalizing problems | -.19** | -.13* | -.12 | -.10 | 1 | .31** | x | x |
| 6. Externalizing problems | -.09 | -.11 | -.04 | -.07 | .37** | 1 | .35** | .21** |
| 7. Depressive symptoms | -.23** | -.13* | -.19** | -.12 | x | .37** | 1 | .78** |
| 8. Anxiety symptoms | -.07 | -.10 | .01 | -.05 | x | .28** | .63** | 1 |
|  |  |  |  |  |  |  |  |  |
|  |  |  |  |  |  |  |  |  |

| Correlations boys (below) and girls (above) Wave 5 |  |  |  |  |  |  |  |  |
| --- | --- | --- | --- | --- | --- | --- | --- | --- |
| Variable | 1 | 2 | 3 | 4 | 5 | 6 | 7 | 8 |
| 1. Father autonomy support: Child-reported | 1 | .29** | .58** | .19* | -.28** | -.25** | -.31** | -.19* |
| 2. Father autonomy support: Father-reported | .22** | 1 | .23** | .28** | -.03 | -.16* | -.04 | -.02 |
| 3. Mother autonomy support: Child-reported | .64** | .19** | 1 | .14 | -.38** | -.29** | -.39** | -.33** |
| 4. Mother autonomy support: Mother-reported | .14* | .21** | .10 | 1 | -.01 | -.03 | -.04 | .04 |
| 5. Internalizing problems | -.03 | -.03 | -.21** | -.01 | 1 | .44** | x | x |
| 6. Externalizing problems | -.13 | -.12 | -.14* | -.05 | .29** | 1 | .45** | .38** |
| 7. Depressive symptoms | -.07 | -.07 | -.20** | -.04 | x | .31** | 1 | .79** |
| 8. Anxiety symptoms | -.02 | .04 | -.18** | .08 | x | .20** | .64** | 1 |
| Correlations boys (below) and girls (above) Wave 6 |  |  |  |  |  |  |  |  |
| Variable | 1 | 2 | 3 | 4 | 5 | 6 | 7 | 8 |
| 1. Father autonomy support: Child-reported | 1 | .22** | .47** | .09 | -.34** | -.27** | -.39** | -.25** |
| 2. Father autonomy support: Father-reported | .26** | 1 | .16* | .12 | -.10 | -.05 | -.12 | -.05 |
| 3. Mother autonomy support: Child-reported | .59** | .23** | 1 | .20** | -.28** | -.22** | -.31** | -.21** |
| 4. Mother autonomy support: Mother-reported | .14* | .18* | .24** | 1 | .04 | -.01 | .01 | .08 |
| 5. Internalizing problems | -.21** | -.06 | -.10 | .02 | 1 | .58** | x | x |
| 6. Externalizing problems | -.04 | -.09 | .00 | .08 | .44** | 1 | .58** | .52** |
| 7. Depressive symptoms | -.27** | -.07 | -.18** | -.01 | x | .42** | 1 | .81** |
| 8. Anxiety symptoms | -.07 | -.03 | .05 | .07 | x | .37** | .64** | 1 |

Appendix D.

*Overview of All Parameter Estimates in the Four Models*

|  | Internalizing problems | |  | Externalizing problems | |  |
| --- | --- | --- | --- | --- | --- | --- |
| Parameters | Child-reported | Parent-reported |  | Child-reported | Parent-reported | |
|  | Beta | Beta |  | Beta | Beta | |
| **Between-Family Associations** |  |  |  |  |  | |
| Father autonomy – mother autonomy | **0.80***** | **0.29***** |  | **0.80***** | **0.28***** | |
| Father autonomy – problem behavior | **-0.44***** | **-0.13**** |  | **-0.40***** | **-0.21**** | |
| Mother autonomy – problem behavior | **-0.39***** | -0.05 |  | **-0.28***** | -0.12* | |
| SES 🡪 father autonomy  SES 🡪 mother autonomy  SES 🡪 problem behavior | **0.73****  0.45*  -0.23 | **0.56****  **0.40****  -0.23 |  | **0.74****  0.45*  -0.35 | **0.53****  **0.41****  -0.33 | |
| **Within-Family Associations** |  |  |  |  |  | |
| T1 father autonomy – mother autonomy | **0.56***** | 0.03 |  | **0.56***** | 0.03 | |
| T1 father autonomy – problem behavior | **-0.25***** | -0.04 |  | **-0.18**** | -0.02 | |
| T1 mother autonomy – problem behavior | -0.12* | -0.09 |  | **-0.18**** | -0.08 | |
| T2-T6 father autonomy – mother autonomy | **0.41*** to 0.54***** | 0.06* to 0.07* |  | **0.41*** to 0.54***** | 0.06 to 0.07* | |
| T2-T6 father autonomy – problem behavior | -0.06* to -0.08* | -0.04 to -0.05 |  | -0.03 to -0.05 | -0.02 to -0.03 | |
| T2-T6 mother autonomy – problem behavior | **-0.08** to -0.10**** | -0.03 to -0.04 |  | -0.05 to -0.07 | 0.02 to 0.02 | |
| **Within-Family Effects** |  |  |  |  |  | |
| Father autonomy 🡪 mother autonomy | 0.04 to 0.04 | -0.00 to -0.00 |  | 0.04 to 0.04 | -0.00 to -0.00 | |
| Mother autonomy 🡪 father autonomy | -0.00 to -0.00 | -0.01 to -0.02 |  | -0.00 to -0.01 | -0.01 to -0.02 | |
| Father autonomy 🡪 problem behavior | 0.03 to 0.04 | 0.03 to 0.03 |  | 0.05 to 0.07 | 0.01 to 0.01 | |
| Mother autonomy 🡪 problem behavior | -0.04 to -0.05 | 0.02 to 0.03 |  | -0.04 to -0.06 | 0.01 to 0.01 | |
| Problem behavior 🡪 father autonomy | 0.00 to 0.00 | -0.01 to -0.01 |  | -0.01 to -0.01 | -0.03 to -0.03 | |
| Problem behavior 🡪 mother autonomy | -0.03 to -0.05 | -0.01 to -0.02 |  | -0.03 to -0.03 | -0.03 to -0.04 | |
| Age 🡪 father autonomy | -0.01 to -0.02 | -0.02 to -0.03 |  | 0.00 to 0.00 | -0.01 to -0.01 | |
| Age 🡪 mother autonomy | -0.05 to -0.05 | -0.07 to -0.08 |  | -0.03 to -0.04 | -0.06 to -0.07 | |
| Age 🡪 problem behavior | -0.03 to -0.05 | -0.03 to -0.04 |  | -0.06* to -0.07* | -0.06* to -0.07* | |
| **Stability Pathways** |  |  |  |  |  | |
| Stability problem behavior | **0.25*** to 0.35***** | **0.25*** to 0.35***** |  | **0.38*** to 0.54***** | **0.38*** to 0.55***** | |
| Stability father autonomy | **0.22*** to 0.28***** | 0.10* to 0.12* |  | **0.22*** to 0.28***** | 0.10* to 0.12* | |
| Stability mother autonomy | **0.14** to 0.16**** | 0.05 to 0.07 |  | **0.14** to 0.16**** | 0.05 to 0.07 | |

*Note.* Boldface coefficients: *p* <.012 (significance level after Bonferroni correction).

* *p* <.05, ** *p* < .01, *** *p* <.001.
